# Supplementary material for: The interactome of intact mitochondria by cross-linking mass spectrometry provides evidence for coexisting respiratory supercomplexes
Source: Mol Cell Proteomics. 2017 Dec 8;17(2):216–32. doi: 10.1074/mcp.RA117.000470 (PMC5795388; doi:10.1074/mcp.RA117.000470)
Supplement: Supplemental Data [file supp_RA117.000470_133922_0_supp_23978_fzffwf.zip › spectra_annotation/mito_DR_spectra_annotation/422-2-8-1-18-1.pdf]

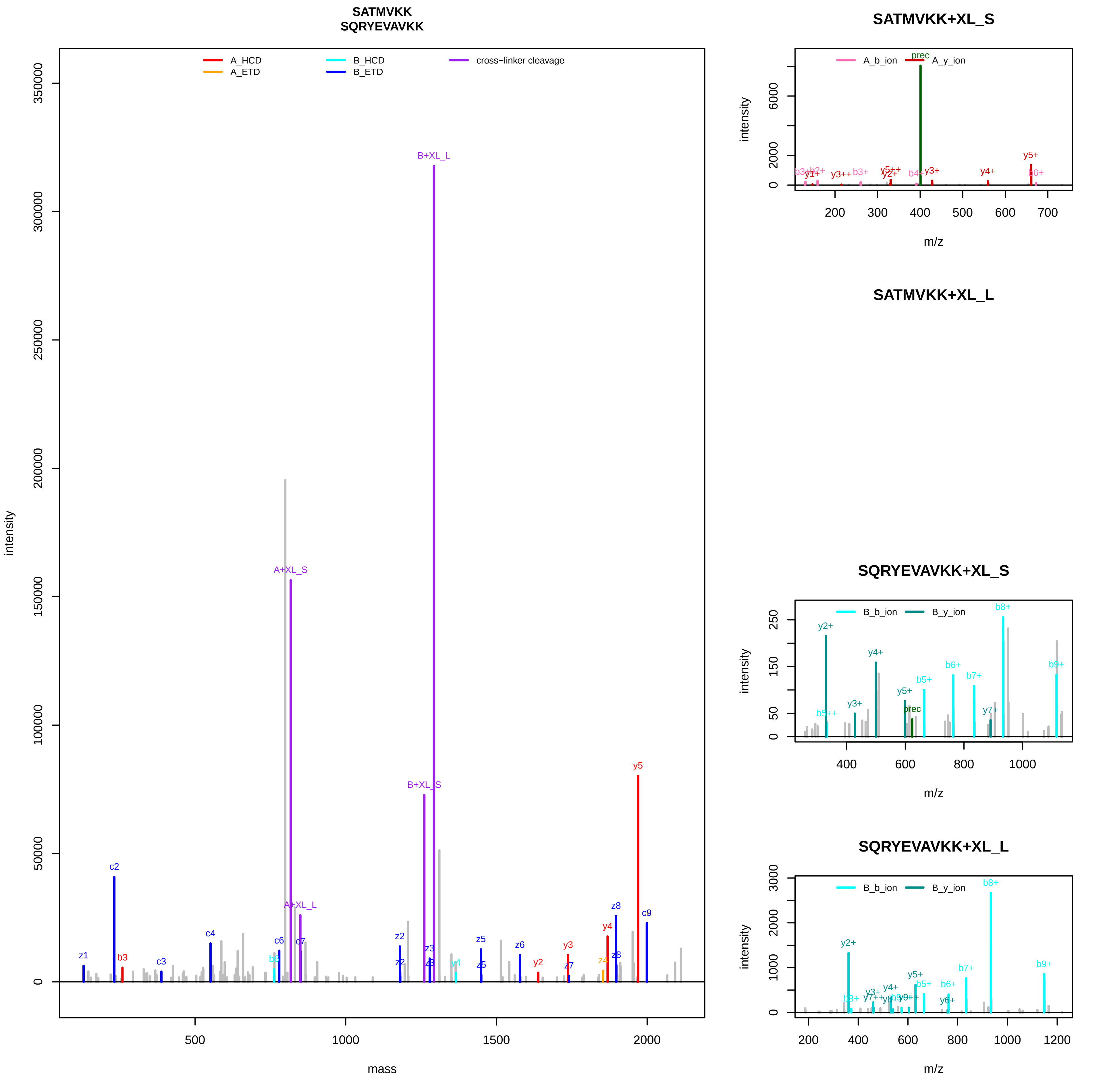
SATMVKK
SQRYEVAVKK
A_HCD
A_ETD
B_HCD
B_ETD
cross−linker cleavage
intensity
mass
0
50000
100000
150000
200000
250000
300000
350000
500
1000
1500
2000
B+XL_L
A+XL_S
B+XL_S
A+XL_L
y5
c2
z1
b3
c3
c4
c6
c7
z2
z3
z5
z6
y2
y3
z7
y4
z4
z8
c9
SATMVKK+XL_S
A_b_ion
A_y_ion
prec
m/z
0
2000
6000
200
300
400
500
600
700
y1+
y3++
b3+
y5++
y2+
b4+
y3+
y4+
y5+
b6+
SATMVKK+XL_L
SQRYEVAVKK+XL_S
B_b_ion
B_y_ion
0
50
150
250
400
600
800
1000
y2+
y4+
b8+
y3+
y5+
prec
b5+
b6+
b7+
y7+
b9+
b5++
SQRYEVAVKK+XL_L
0
1000
2000
3000
200
400
600
800
1000
1200
y2+
y3+
y4+
y5+
b5+
b6+
b7+
b8+
b9+
y6+
b3+
